# Detection of new SHV-12, SHV-5 and SHV-2a variants of extended spectrum Beta-lactamase in *Klebsiella pneumoniae* in Egypt

**DOI:** 10.1186/1476-0711-12-16

**Published:** 2013-07-18

**Authors:** Enas A Newire, Salwa F Ahmed, Brent House, Esmeralda Valiente, Guillermo Pimentel

**Affiliations:** 1 Global Disease Detection and Response Program, U.S. Naval Medical Research Unit No. 3 (NAMRU-3), Abbassia, Cairo, Egypt; 2 Department of Pathogen Molecular Biology, London School of Hygiene and Tropical Medicine, Keppel Street, London, UK

## Abstract

**Background:**

*Klebsiella pneumoniae* outbreaks possessing extended-spectrum β-lactamase- (ESBL) mediated resistance to third-generation cephalosporins have increased significantly in hospital and community settings worldwide. The study objective was to characterize prevalent genetic determinants of TEM, SHV and CTX-M types ESBL activity in *K. pneumoniae* isolates from Egypt.

**Methods:**

Sixty five ESBL-producing *K. pneumoniae* strains, isolated from nosocomial and community-acquired infections from 10 Egyptian University hospitals (2000–2003), were confirmed with double disc-synergy method and E-test. *bla*TEM, *bla*SHV and *bla*CTX-m genes were identified by PCR and DNA sequencing. Pulsed-field gel electrophoresis (PFGE) was conducted for genotyping.

**Results:**

All isolates displayed ceftazidime and cefotaxime resistance. *bla*TEM and *bla*SHV genes were detected in 98% of the isolates’ genomes, while 11% carried *bla*CTX-m. DNA sequencing revealed plasmid-borne SHV-12,-5,-2a (17%), CTX-m-15 (11%), and TEM-1 (10%) prevalence. Among SHV-12 (n=8), one isolate displayed 100% blaSHV-12 amino acid identity, while others had various point mutations: T17G (Leu to Arg, position 6 of the enzyme: n=2); A8T and A10G (Tyr and Ile to Phe and Val, positions 3 and 4, respectively: n=4), and; A703G (Lys to Glu 235: n=1). SHV-5 and SHV-2a variants were identified in three isolates: T17G (n=1); A703G and G705A (Ser and Lys to Gly and Glu: n=1); multiple mutations at A8T, A10G, T17G, A703G and G705A (n=1). Remarkably, 57% of community-acquired isolates carried CTX-m-15. PFGE demonstrated four distinct genetic clusters, grouping strains of different genetic backgrounds.

**Conclusions:**

This is the first study demonstrating the occurrence of SHV-12, SHV-5 and SHV-2a variants in Egypt, indicating the spread of class A ESBL in *K. pneumoniae* through different mechanisms.

## Background

*K. pneumoniae* is a facultative anaerobic, Gram negative bacteria of the Enterobacteriaceae family, and a reported opportunistic pathogen that has been implicated in many community- and hospital-acquired infections [1]. This organism can initiate urinary tract infections, wound infections, pneumonia, intra-abdominal infections, nasal mucosa atrophy, and rhinoscleroma. The number of outbreaks involving *K. pneumoniae* strains with extended spectrum β-lactamase (ESBL) mediated resistance to third-generation cephalosporins has been progressively increasing in many parts of the world [1].

β-lactamases, the precursors to ESBLs, confer resistance by inactivating β-lactam antibiotics [2], like penicillins, cephalosporins, carbapenems, and monobactams, by breaking open the four atom β-lactam ring structure. The *extended spectrum* β-lactamases (ESBLs) are a rapidly developing group of enzymes with the ability to hydrolyze third-generation cephalosporins as well as the monobactams and aztreonams that are known to be active against *Klebsiella* species. Clavulanic acid is a potent inhibitor of the β-lactamases and is commonly included with β-lactam antibiotics when an ESBL-producing bacterium is suspected. Clavulanic acid is a member of the clavams or oxapenams that inhibits β-lactamase activity by cova-lently binding to the serine that resides in the active site of the enzyme.

ESBL genes originally evolved from the β-lactamase TEM-1, TEM-2, and SHV-1 genes through mutations of the amino acids surrounding the active site β-lactamases.

ESBLs have recently become a significant problem because they are commonly plasmid-encoded, facilitating a high rate of horizontal transfer between different bacterial species [3]. Furthermore, such plasmids typically carry resistance genes to other drugs such as the aminoglycosides, thus narrowing treatment options.

As an illustration, administration of cephalosporins has recently been linked to increasing treatment failure rates, and isolated strains have been found to possess higher minimal inhibitory concentration (MIC) rates for ceftriaxone, cefotaxime and, to a lesser extent, ceftazidime. However, this resistance diminishes in the presence of β-lactamase inhibitors, such as clavulanic acid [4]. This type is considered class-A ESBL resistance, where the TEM, SHV and CTX-M type ESBL enzymes are able to hydrolyze [5].

Previous molecular characterization studies on TEM, SHV, and CTX-m genes and their derivatives, e.g. *bla*TEM-1 and *bla*SHV-12, have shown that they are epidemiologically related, and are both plasmid-borne [6]. PFGE have also been widely employed to investigate the epidemiological and genotypic relatedness of ESBL-producing bacteria [1] and to track evolving strains implicated in various geographic regions [6]. Recent studies in the Middle East have revealed a high prevalence of ESBL-producing *K. pneumoniae* isolates in Israel [7], and extensive spread of those carrying CTX-m-15 was reported in Lebanon, Kuwait, and Egypt [8-10]. In Saudi Arabia, CTX-M-15 producing *K. pneumoniae* was responsible for a neonatal intensive care unit outbreak [11].

Our goal was to characterize and determine the prevalence of genetic elements of ESBL-producing *K. pneumoniae* collected between 2000 and 2003 in Egypt. Isolates in this study including those from both community-acquired and nosocomial infections, were genotyped and their DNA fingerprints were compared.

## Methods

### Bacterial isolates

A total of 65 *K. pneumoniae* were isolated from blood specimens collected as part of an infection control surveillance study carried out by the Infection Control Unit, Global Disease Detection and Response Program (GDDRP), U.S. Naval Medical Research Unit No. 3 (NAMRU-3), Cairo, Egypt, in partnership with the U.S. Centers for Disease Control and Prevention (CDC) (Table 1). Isolates were collected from 10 different Egyptian teaching hospitals distributed across seven governorates in Egypt: Cairo, Alexandria, Suez, Sohag, Dakahlia, Sharkiya, and Kafr El-Sheikh. Microbiological isolation and biochemical identification procedures were conducted according to Clinical and Laboratory Standards Institute procedures (CLSI) [12].

**Table 1:** Strain information

**No**	**Isolate accession**	**Hospital site**	**ESBL test**	**Type of infection**	**CTX-M Plasmid**	**TEM Plasmid**	**SHV Plasmid**	**CTX-M Chrom**	**TEM Chrom**	**SHV Chrom**
1	00-008416	Cairo Univ	**+**	**Community**	**-**	**-**	**-**	**-**	**+**	**+**
2	01-001425	Cairo Univ	**+**	**Community**	**-**	**-**	**-**	**-**	**+**	**+**
3	01-007549	Cairo Univ	**+**	Nosocomial	**-**	**-**	**-**	**-**	**+**	**+**
4	01-007550	Cairo Univ	**+**	Nosocomial	**-**	**-**	**-**	**-**	**+**	**+**
5	01-007724	Cairo Univ	**+**	Nosocomial	**-**	**+**	**+**	**-**	**+**	**+**
6	01-007732	Cairo Univ	**+**	Nosocomial	**-**	**-**	**-**	**-**	**+**	**+**
7	01-007743	Cairo Univ	**+**	Nosocomial	**-**	**+**	**+**	**-**	**+**	**+**
8	01-007949	Cairo Univ	**+**	Nosocomial	**-**	**-**	**-**	**-**	**+**	**+**
9	01-007951	Cairo Univ	**+**	Nosocomial	**-**	**+**	**+**	**-**	**+**	**+**
10	01-007953	Cairo Univ	**+**	Nosocomial	**-**	**-**	**-**	**-**	**+**	**+**
11	01-007959	Cairo Univ	**+**	Nosocomial	**-**	**+**	**-**	**-**	**+**	**+**
12	01-007961	Cairo Univ	**+**	Nosocomial	**-**	**-**	**-**	**-**	**+**	**+**
13	01-007967	Cairo Univ	**+**	Nosocomial	**-**	**-**	**-**	**-**	**-**	**-**
14	01-008663	Cairo Univ	**+**	Nosocomial	**-**	**-**	**-**	**-**	**+**	**+**
15	01-008664	Cairo Univ	**+**	Nosocomial	**-**	**-**	**-**	**-**	**+**	**+**
16	01-008666	Cairo Univ	**+**	Nosocomial	**-**	**-**	**+**	**-**	**+**	**+**
17	01-008671	Cairo Univ	**+**	Nosocomial	**-**	**-**	**-**	**-**	**+**	**+**
18	01-008672	Cairo Univ	**+**	Nosocomial	**-**	**-**	**-**	**-**	**+**	**+**
19	01-008884	Cairo Univ	**+**	Nosocomial	**-**	**-**	**-**	**-**	**+**	**+**
20	01-009044	Cairo Univ	**+**	Nosocomial	**-**	**-**	**-**	**-**	**+**	**+**
21	01-012909	Cairo Univ	**+**	Nosocomial	**-**	**-**	**-**	**-**	**+**	**+**
22	01-012910	Cairo Univ	**+**	Nosocomial	**-**	**-**	**-**	**-**	**+**	**+**
23	01-012912	Cairo Univ	**+**	Nosocomial	**-**	**-**	**-**	**-**	**+**	**+**
24	01-012919	Cairo Univ	**+**	Nosocomial	**-**	**-**	**-**	**-**	**+**	**+**
25	01-012921	Cairo Univ	**+**	Nosocomial	**-**	**+**	**-**	**-**	**+**	**+**
26	01-012925	Cairo Univ	**+**	Nosocomial	**-**	**-**	**-**	**-**	**+**	**+**
27	01-013098	Cairo Univ	**+**	Nosocomial	**-**	**-**	**-**	**-**	**+**	**+**
28	01-013099	Cairo Univ	**+**	Nosocomial	**-**	**-**	**-**	**-**	**+**	**+**
29	01-013638	Cairo Univ	**+**	Nosocomial	**-**	**-**	**-**	**-**	**+**	**+**
30	01-013639	Cairo Univ	**+**	Nosocomial	**-**	**-**	**-**	**-**	**+**	**+**
31	01-013640	Cairo Univ	**+**	Nosocomial	**-**	**-**	**-**	**-**	**+**	**+**
32	01-013906	Cairo Univ	**+**	Nosocomial	**-**	**-**	**+**	**-**	**+**	**+**
33	01-015467	Cairo Univ	**+**	Nosocomial	**-**	**-**	**-**	**-**	**+**	**+**
34	01-015468	Cairo Univ	**+**	Nosocomial	**-**	**-**	**-**	**-**	**+**	**+**
35	01-015469	Cairo Univ	**+**	Nosocomial	**+**	**-**	**-**	**+**	**+**	**+**
36	01-015470	Cairo Univ	**+**	Nosocomial	**+**	**-**	**-**	**+**	**+**	**+**
37	01-015472	Cairo Univ	**+**	Nosocomial	**-**	**-**	**-**	**-**	**+**	**+**
38	01-015473	Cairo Univ	**+**	Nosocomial	**-**	**-**	**-**	**-**	**+**	**+**
39	01-015474	Cairo Univ	**+**	Nosocomial	**-**	**-**	**-**	**-**	**+**	**+**
40	01-015475	Cairo Univ	**+**	Nosocomial	**-**	**-**	**-**	**-**	**+**	**+**
41	01-015857	Cairo Univ	**+**	Nosocomial	**-**	**-**	**-**	**-**	**+**	**+**
42	01-015858	Cairo Univ	**+**	Nosocomial	**-**	**-**	**-**	**-**	**+**	**+**
43	01-015859	Cairo Univ	**+**	Nosocomial	**-**	**-**	**-**	**-**	**+**	**+**
44	01-015860	Cairo Univ	**+**	Nosocomial	**-**	**-**	**-**	**-**	**+**	**+**
45	01-016401	Cairo Univ	**+**	Nosocomial	**-**	**-**	**+**	**-**	**+**	**+**
46	01-017000	Cairo Univ	**+**	Nosocomial	**-**	**-**	**-**	**-**	**+**	**+**
47	01-017852	Cairo Univ	**+**	Nosocomial	**-**	**-**	**-**	**-**	**+**	**+**
48	01-017853	Cairo Univ	**+**	Nosocomial	**-**	**-**	**-**	**-**	**+**	**+**
49	01-017866	Cairo Univ	**+**	Nosocomial	**-**	**-**	**-**	**-**	**+**	**+**
50	01-018867	Zagazig Univ	**+**	Nosocomial	**-**	**-**	**+**	**-**	**+**	**+**
51	02-000429	Zagazig Univ	**+**	**Community**	**-**	**-**	**+**	**-**	**+**	**+**
52	02-000569	Zagazig General Hospital	**+**	Nosocomial	**-**	**-**	**-**	**-**	**+**	**+**
53	02-005285	Mansoura General Hospital	**+**	Nosocomial	**+**	**-**	**-**	**+**	**+**	**+**
54	02-005864	Sohag General Hospital	**+**	Nosocomial	**-**	**-**	**-**	**-**	**+**	**+**
55	02-009772	Suez General Hospital	**+**	Nosocomial	**-**	**-**	**-**	**-**	**+**	**+**
56	02-009785	Sohag General Hospital	**+**	Nosocomial	**-**	**-**	**-**	**-**	**+**	**+**
57	02-010707	Fawzi Moaz General Hospital	**+**	Nosocomial	**-**	**-**	**-**	**-**	**+**	**+**
58	02-010718	Mansoura General Hospital	**+**	Nosocomial	**-**	**-**	**-**	**-**	**+**	**+**
59	02-018404	Suez General Hospital	**+**	Nosocomial	**-**	**-**	**-**	**-**	**+**	**+**
60	03-000024	Kafr El Shiek General	**+**	Nosocomial	**-**	**-**	**-**	**-**	**+**	**+**
61	03-002582	Kafr El Shiek General Hospital	**+**	Nosocomial	**-**	**-**	**+**	**-**	**+**	**+**
62	03-018785	Kafr El Shiek General Hospital	**+**	**Community**	**+**	**-**	**+**	**+**	**+**	**+**
63	03-021318	Kafr El Shiek General Hospital	**+**	**Community**	**+**	**-**	**-**	**+**	**+**	**+**
64	03-021320	Kafr El Shiek General Hospital	**+**	**Community**	**+**	**-**	**-**	**+**	**+**	**+**
65	03-021322	Kafr El Shiek General Hospital	**+**	**Community**	**+**	**+**	**+**	**+**	**+**	**+**

Isolates accessioning numbers utilized in the study are shown above: The first two digits indicate the year of collection. The last six columns indicate the presence (+) or absence (-) of the genes tested on both chromosomal and plasmid. Community source of infection is distinguished in bold.

### Antibiotic susceptibility testing and ESBL detection

Antibiotic susceptibility was determined by the agar disk diffusion method. The following antibiotics were tested: ampicillin (30 μg), imipenem (10 μg), cefepime (30 μg), cephalothin (30 μg), ceftriaxone (30 μg), cefpodoxime (10 μg), ceftazidime (30 μg), cefotaxime (30 μg), and sulfamethoxazole (30 μg) (Becton Dickinson, Sparks, MD, USA), with results interpreted using the CLSI criteria [12]. Also, minimum inhibitory concentration (MIC) was evaluated by E-test for ceftazidime, cefepime, ceftriaxone, and cephalothin (bioMérieux, France) [13]. Additionally, the detection of ESBL activity was screened by double-synergy test [14]. *K. pneumoniae* ATCC 700603 and *E. coli* ATCC 25922 were used as positive and negative controls, respectively, as recommended by CLSI guidelines for Enterobacteriaceae [12].

### Pulsed-field gel electrophoresis (PFGE)

*K. pneumoniae* isolates were subjected to PFGE. Total DNA was extracted and digested using the *Xba1*restriction enzyme following the procedures described in the PulseNet International protocols for *Salmonella* with minor modifications pertaining to electrophoresis running conditions in the CHEF-DR III PFGE system (BioRad, Hercules, USA): 19 hrs, with initial switch time 2.2 sec, final switch time 63.8 sec, at 6 volts, and at 120˚ Angle [15].

### DNA extraction (chromosomal and plasmid)

Twenty-four hour culture of *K.pneumoniae* isolates grown on MacConkey Agar were used for DNA extraction. Chromosomal and plasmid DNA extractions were performed using the Qiagen Blood Mini Kit (QIAgen Inc., Valencia CA, USA), and Wizard plus Minipreps SV DNA purification system (Promega, Madison, USA), respectively, according to the manufacturer’s instructions. The quality of the extracted chromosomal and plasmid DNA was confirmed by running the extracts on 0.8% agarose gel (100 Volts, 400 amps, for 2 hrs), and by measuring the concentration of DNA by NanoDrop (Thermo Scientific, Kansas, USA).

### PCR screening for ESBL genes

Both chromosomal and plasmid DNA extracts of all isolates were screened by PCR for the presence of three ESBL genes: TEM, SHV, and CTXm. Three sets of primers were designed for screening (Table 2): a core set to amplify the conserved, central region of each gene for an initial screening [16]; two sets of degenerate primers to detect the more variable upstream and downstream regions of each gene for confirmation screening. PCR primers were purchased commercially (Sigma-Genosys, Taufkirchen, Germany). Five μl of the DNA template were added to complete a final PCR reaction volume of 25 μl containing: 1× green reaction buffer; 3 mM of MgCl2.

**Table 2:** PCR and DNA primers sequencing of the three ESBL genetic markers

**Primer**	**Sequence**	**Size (bp)**	**Reference**
Core TEM F	CAGCGGTAAGATCCTTGAGA	643	[9]
Core TEM R	ACTCCCCGTCGTGTAGATAA		
Core SHV F	GGCCGCGTAGGCATGATAGA	714	
Core SHV R	CCCGGCGATTTGCTGATTTC		
Core CTXM F	AACCGTCACGCTGTTGTTAG	766	
Core CTXM R	TTGAGGCGTGGTGAAGTAAG		
Upstream TEM F Upstream TEM R	TGAAGACGAAAGGGCCTCCTG ACTCCCCGTCGTGTAGATAA	109	Newly designed primers in GDDRP Molecular Laboratoryfor this study
Upstream SHV F Upstream SHV R	CGTTWTDTTCGCCKGTGT CGAGTAGTCCACCAGATCCTG	74	
Upstream XTXM-F Upstream CXTXM-R	ATGGTTAAAAAATCACTG ACGTTATCGCTGTACTGTA	55	
Downstream TEM F Downstream TEM R	CAGCGGTAAGATCCTTGAGA TAATCAGTGAGGCACCTATCTC	109	
Downstream SHV F Downstream SHV R	GGCCGCGTAGGCATGATAGA CCCGGCGATTTGCTGATTTC	74	
Downstream CTXM-F Downstream CTXM-R	TACAGTACAGCGATAACGTGG CCGTTTCCGCTATTACAAA	55	

(Promega, Madison, USA); 0.3 pM of each primer; 2.5U of GoTaq Flexi DNA Polymerase and 0.4 mM dNTPs. Template amplification was accomplished using the following cycle conditions: 95°C for 5 minutes (initial denaturation),30 cycles of: 94°C for 30 sec (denaturation), 52°C for 45 sec (annealing), and 72°C for 45 sec (extension), 72°C for 7 min (final extension). The annealing temperatures and times for TEM, SHV and CTXm were 52°C for 45 sec, 55°C for 1 min, and 57°C for 45 sec, respectively.

PCR products were visualized on Ethidium-stained 1% agarose gels with expected product lengths of 643 bp, 714 bp and 766 bp for *bla*TEM, *bla*shv, and *bla*CTX-m, respectively.

### ESBL gene sequencing

After initial screening for the amplification of core ESBL genes on both chromosomal and plasmid, the plasmid-borne TEM, SHV, or CTX-m genes were subjected to nucleic acid sequencing.

The initial PCR amplified products were purified and treated with Exo Sap-it enzyme (USB, Santa Clara CA, USA) at the ratio of 2:1 using 10 μl of the amplicon to 5 μl of the enzyme. Each mixture was incubated in a conventional thermal cycler (Applied Biosystems, CA, USA) for 15 min at 37°C, followed by 15 min at 80°C according to the manufacturer’s instructions. Direct sequencing of each amplicon was carried out using the Sanger dideoxynucleotide chain termination method with the ABI Prism Big Dye Terminator Cycle Sequencing Reaction Kit (Applied Biosystems, Inc., Foster City, CA, USA) on an ABI Prism 3130 Automated Sequencer. Using data collection software version 2.0, and sequencing analysis software 5.1.1.

For each sequencing reaction, 2 μl purified PCR product were added to a final reaction volume of 20 μl containing 1× of sequencing buffer (Applied Biosystems, CA, USA); 4 μl BigDye (Applied Biosystems, USA) reaction mix; and 1.5pM of each of the Forward and Reverse primer. The sequencing cycle was composed of two stages; stage one is denaturing at 96°C for 10 sec, while stage two is composed of 25 cycles of denaturing at 96°C for 10 sec, annealing at 50°C for 5 sec, and extension at 60°C for 4 min.

Each cycle sequence product was purified by spin column purification according to the manufacturer’s instructions (Edge Biosystems, CA USA). The purified PCR product was then completely dried at 85°C for 30–40 min. Fifteen μl Hi-Di formamide (Applied Biosystems, CA, USA) was added to the tubes, after which the tubes were heated for 2 min at 95°C, then immediately placed on ice.

### Data analyses

Nucleotide sequences obtained from various isolates were assembled using the software programs BioNumerics (version 5.10) and BioEdit (version 7.0.9.0), and aligned with GenBank reference sequences using the Clustal X application within BioEdit. Translated amino acid sequences were compared with published sequences in the Lahey organization (http://www.lahey.org/studies). Phylogenetic tree was generated using the MEGA version 4 software, and dendrogram were constructed using the neighbor-joining method [15]. PFGE data from the 65 isolates was analyzed using BioNumerics (version 5.10). Genetic similarities were inferred from dendrograms created using the unweighted pair-group method with average (UPGMA) analysis of the XbaI mrp–PFGE patterns using the Dice coefficient with a 1.5% tolerance for the band migration distance.

## Results

### Antibiograms of *K. pneumoniae* isolates

In hospitalized and community cases, 100% of the strains in this study have shown susceptibility in the presence of β-lactam inhibitor CAZ/CLA, while 98.5% (64/ 65) were susceptible to CTX/CLA excluding one isolate (01–007953) with inhibition zone diameter ≥ 5 mm. Variable resistance patterns were seen among other antibiotics (Table 3). All *K. pneumoniae* isolates displayed resistance to ampicillin(AM), ceftazidime (CAZ), cephalothin (CE), ceftriaxone (CF), and cefpodoxime (CPD), while the majority of isolates, 97%, were resistant to cefotaxime (CTX). On the other hand, the isolates displayed lower rates of resistance to sulfamethoxazole (SXT), cefepime (FEP), (PM), and imipenem (IPM) (49%, 37%, 14%, and 3%, respectively).

**Table 3:** Antibiotic resistance profile

	**Panel of antibiotic tested**													
	Disk diffusion*						**ESBL Confirmation†**				**E-test‡**			
	**Ampicillin (AM)**	**Imipenem (IPM)**	**Cefepime (FEP)**	**Cephalothin (CF)**	**Ceftriaxone (CRO)**	**Cefpodoxime (CPD)**	**CAZ**	**CAZ/CLA ZONE**	**CTX**	**CTX/CLA zone**	**Sulfamethoxazole (SXT)**	**Ceftazidime (TZ)**	**Cefepime (PM0**	**Cephalothin (CE)**
**N(%) Cases with resistance phenotype**	65 (100)	1 (3)	24 (37)	65 (100)	61 (94)	65 (100)	65 (100)	0 (0)	63 (97)	1 (1.5)	32 (49)	64 (98)	9 (14)	65 (100)

The table is divided into three major categories to show the number and percent [N (%) ] of cases with ESBL phenotype by Disc diffusion*, double disc synergy test (for ESBL confirmation)† and E test results ‡ to SXT and other third generation antibiotics.

### Molecular characterization of ESBL genes

Amplification and sequencing primers were designed to both detect the presence of and generate templates for the accurate sequencing of the relatively large genes studied here (Table 2).

Eleven percent of isolates (7/65) carried the CTX-m gene. The majority (4/7) of the community isolates carried CTX-m, compared to only 5% (3/58) of the nosocomial isolates. Amplification of the upstream and downstream regions and sequencing of the full length PCR products (766 bp core region and 55 bp upstream and downstream regions: 876 bp total) confirmed that the seven plasmid-borne CTX-m genes were 100% identical to CTX-m-15 reported in GenBank.

Ninety-eight percent (64/65) of the total isolates also carried the SHV core gene chromosomally. Of note, the remaining single isolate (01–007967, Table 1) was negative for the three studied genetic markers. Seventeen percent (11/65) of isolates carried SHV on the plasmid. Four of the eleven isolates carried both the TEM and SHV genes on a plasmid, while no plasmid carriage was detected in the remaining seven isolates.

DNA sequencing analysis of whole SHV gene from 11 isolates revealed 99% similarity to SHV-12. On the nucleotide level, no similarity was found between the eleven SHV gene sequences to each other or published SHV sequences: SHV-12 (GenBank# GU064393.1), and the closely related SHV-5 (GenBank# HM125696.1), and SHV-2a (GenBank# GQ407116.1). Comparison of the corresponding amino acid sequence of each gene utilizing Lahey organization study tables, demonstrated SHV-12, SHV-5 and SHV-2a as the most closely related sequencing match (Table 2). Also, see Additional file 1 PDF displays SHV amino acid sequence variations of ESBL Egyptian isolates table.

### Macro-restriction pattern typing using Pulsed-Field Gel Electrophoresis (PFGE)

**Figure 1:**
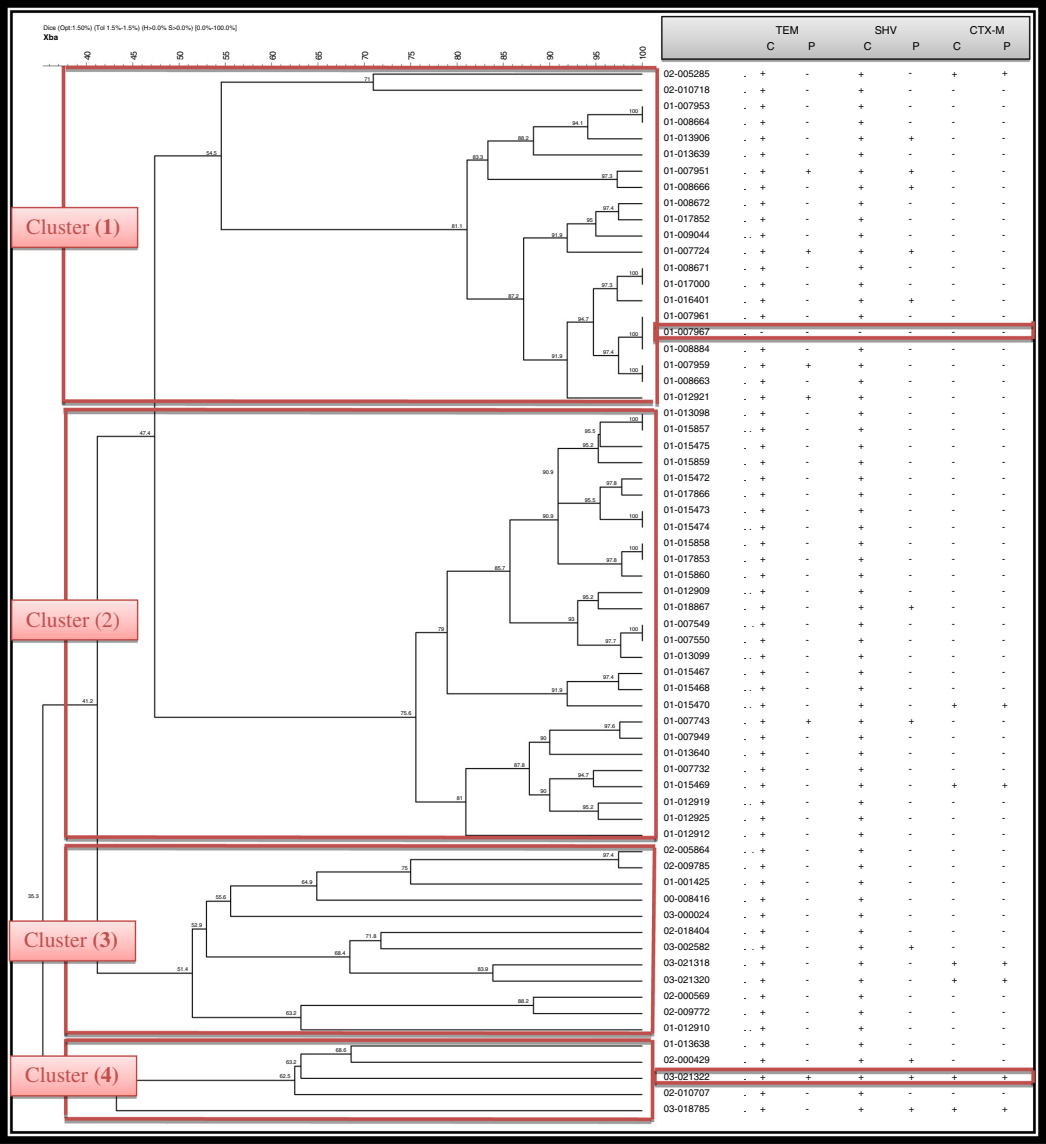
**Phylogenetic tree of Egyptian ESBL isolates (n = 65) demonstrating different clusters by PFGE.** C (Chromosomal), and P (Plasmid).

DNA fingerprint profiling of the 65 isolates identified four major clusters (Figure 1), with strains grouped in each cluster sharing high levels of similarity (80-100%).

PFGE identified four major clusters: cluster one grouped 32% of the samples (n=21) with 54.5% similarity. Five of the eleven (45%) plasmid-borne SHV were identified in this cluster, and their mrp were indistinguishable. Remarkably, one isolate (01–007967) did not carry any of the genes, displayed identical MRP to two isolates (01–007961, and 01–008884) carrying both TEM and SHV. Cluster two grouped the majority 41.5% of the isolates (n= 27), sharing 75.6% similarity and forming a broad cluster. Two plasmid-borne SHV and another two CTX-m carrying isolates were identified in this cluster and located distantly from each other. The third cluster is composed of 19% of the samples (n=12), members of this cluster did not share identical MRP pattern. The fourth cluster represented the fewer number of samples 8% (n=5) where one isolate (03–021322) carried the three genetic markers for resistance.

**Figure 2:**
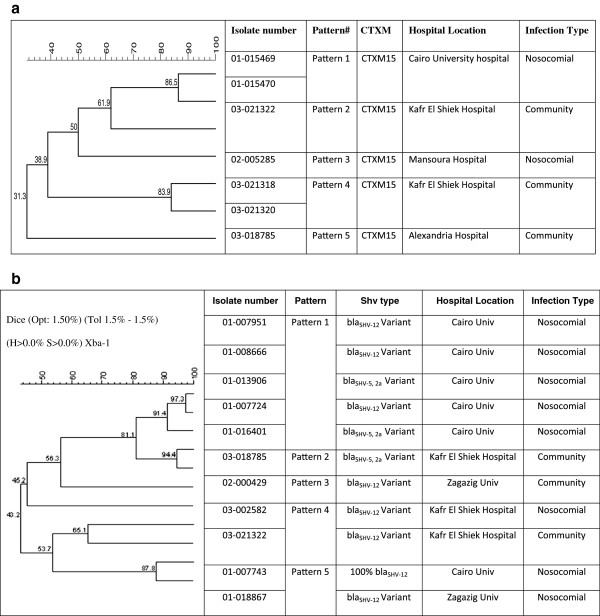
**PFGE Analysis. 2a**. PFGE analysis of the seven CTXM-15 *K. pneumoniae* producers strains. The dendrogram was constructed with dice coefficient, complete linkage, and 1.5% position tolerance. The degree of relatedness between *K. pneumoniae* isolates and those from GenBank is shown on the branches. **2b**. PFGE analysis of the 11 *K. pneumoniae* strains producing SHV-12, SHV5 and SHV-2a. The dendrogram was constructed with dice coefficient, complete linkage, and 1.5% position tolerance. The degree of relatedness among *K. pneumoniae* isolates and those from GenBank is shown on the branches.

PFGE analysis of the seven *K. pneumoniae* strains producing CTX-m-15 (Figure 2), using complete linkage, dice band-based similarity coefficient and 1.5% position tolerance, revealed 5 clusters: one pair of isolates (pattern 1) was collected from nosocomial infections at a hospital in Cairo in 2001 (01–015469 and 01–015470; >85% similarity). Another pair (pattern 4) were isolated in Kafr El Shiek governorate from community-acquired infections in 2003 (03–021318 and 03–021320, > 80% similarity) displayed a MRP (pattern 3) that is distantly related to another isolate detected at the same hospital (03–021322, 61.9% similarity). Two isolates each with unique pattern; one was isolated from Mansoura governorate (nosocomial, 02–005285) formed pattern 4), and another isolate from Alexandria governorate (community, 03–018785) formed pattern 5. Five distinct DNA fingerprint patterns were detected with the positive plasmid SHV isolates (Figure 2b). Interestingly, pattern 1, composed of five isolates (01–007951, 01–008666, 01–013906, 01–007724, 01–016401), all were isolated from nosocomial infection collected from Cairo University. The unique MRP patterns 2 and 4, displayed by isolates 03–018785, and 03–021322, respectively, were collected from Kafr El-Shiek General Hospital but was not seen in the seven nosocomial isolates received from the same location (03–002582). Distinct patterns 3 and 5 came from community isolates collected at Zagazig University.

## Discussion

### Antibiograms of *K. pneumoniae* isolates

Infections caused by ESBLs producing bacteria have become an emerging global problem. Studies on Enterobacteriaceae isolates from Egypt have reported a resistance rate to third generation cephalosporins of 70% [17,18]. A survey, carried out in 2001–2002 which covered medical centers in northern and southern European countries, Egypt, Lebanon, Saudi Arabia and South Africa, reported the highest incidence of extended spectrum β-lactamases (ESBLs)-producing isolates in Egypt [3]. A recent study from Egypt reported an outbreak in a neonatal intensive care unit in Cairo, Egypt, in which 80% of the isolates were *K. pneumoniae*, of which 58% were ESBL producers [19]. While the phenotypic characteristics of ESBL of enterobactericeae were widely studied in Egypt, there were very few published reports on the molecular aspect underlying mechanism of ESBL. In this study, we investigated the genetic determinants of ESBL activity in *K. pneumoniae* isolates collected from 10 different Egyptian teaching hospitals distributed across seven governorates in Egypt.

The ESBL activity of *K. pneumoniae* clinical isolates collected from both community and hospital settings is concordant with previous reports from Egypt and other countries which indicate faecal carriage of these pathogens in the community and may have important implications as a risk factor for acquiring ESBL-producing *K. pneumoniae* isolates upon hospital admission. However, the variable level of resistance of *K. pneumoniae* to other non-β-lactam antibiotics, e.g., sulfamethoxazole data, is not concordant with previous reports from Egypt, where a high level of resistance was found [20]. The difference in sulfamethoxazole resistance may be attributed to a wide dissemination of different ESBL clones of *K. pneumoniae* among different community and hospital settings.

### Molecular characterization of ESBL genes

CTX-M is the most prevalent gene in ESBL-producing enterobacteriaceae worldwide [6]. Recently, CTX-M ESBLs have been reported in different studies in Egypt [10,11,20]. Previous studies have shown CTX-m-15 to be the most commonly reported ESBL type in the Middle East and North Africa [8,20]. In this study, however, the most common ESBL type identified in Egyptian *K. pneumoniae* isolates was SHV (98%), compared to the relatively low prevalence of CTX-m-15 (11%). This differs from a recent report from Egypt indicating that CTX-M-14 is the mechanism of resistance mediating the high resistance of β lactamases *K. pneumoniae* producers to third generation Cephalosporins among resistant clinical isolates at a medical institution in Cairo, Egypt [20]. This may indicate that multiple clones of circulating *K. pneumonia* are involved in the dessimination of high ESBL resistance in both community and hospital settings. Though this study detected a relatively small number (11%) of isolates carrying the CTX-m-15 type of ESBL, it supports our previous report from Egypt indicating the higher prevalence of CTX-m ESBL-producers in *E. coli* than in *K. pneumoniae*[10]. Nevertheless, resistance has been maintained and is sprea-ding regardless of the use of extended-spectrum cephalosporins in metropolitan areas of Egypt [21]. The evidence of possible outbreaks involving CTX-m-15 warrants the implementation of strict hospital infection control policies, including the review of current therapeutic modalities, control of the use of non-prescribed antibiotics, and continuous monitoring of antibiotic sensitivity profiles of *K. pneumoniae* isolates.

As TEM is the primary genetic element implicated in the evolution of ESBL resistance mechanisms [22], we tested for this β-lactamase type. Ninety eight percent (64/65) of current isolates carried the TEM core gene on the chromosome, while only nine percent (6/65) carried the gene on a plasmid (Table 1). Amplification and DNA sequencing of the six plasmid-borne genes revealed 100% identity to the TEM-1 gene reported in GenBank (EU979561.1). Even though TEM 1 is not considered an *extended-spectrum* β-lactamase, it may contribute to this extended spectrum of resistance in concert with other β-lactamases [4].

It is worth mentioning that isolate 01–007967 did not carry TEM, SHV, or CTX-M genes either on the chromosome or on a plasmid. It is likely that this isolate carries one or more β-lactamase genes not tested in this study. It is also possible that any number of the other isolates carried additional undetected β-lactamase genes that conferred the phenotypic high antimicrobial resistance seen.

Comparison of the corresponding amino acid sequence of the 11 SHV positive isolates demonstrated the predominance of SHV-12, while SHV-5 and SHV-2a was the most closely related sequencing match in the remaining isolates. SHV-12 is thought to have evolved from SHV-2a, and SHV-5 from SHV-2 [8]. In comparison, SHV-12 contains only one amino acid (AA) difference at position 31: SHV-12 possesses a Glutamine, while both SHV-2a and SHV-5 possess a Leucine. Upon blasting the nucleic acid sequence of the eleven plasmid-borne SHV genes against GenBank entries, our collection displayed a matching similarity value of 99% to SHV-12 and the closely related SHV-2a and SHV-5 genes [23]. Our phylogenetic tree analysis illustrated the relatedness among the *K. pneumoniae* isolates and SHV-12 (GU064393.1, *K*. *pneumoniae* strain HB 52 β -lactamase blaSHV-12 isolated in Brazil), SHV-2a (GQ407116.1, *K. pneumoniae* strain HB 34 β -lactamase blaSHV-2a isolated in Brazil), and SHV-5 (HM125696.1, *K. pneumoniae* strain S-486 β -lactamase SHV-5 isolated in Russia) GeneBank isolates, enabling to conclude that these genes are globally circulated.

Among the SHV carrying isolates, polymorphisms were seen at position 31 (Q or L), position 6 (R or L), positions 234–235 (SK, GE or SE), and positions 3–4 (YI or FV) of the enzyme. Of particular interest, four variants possessed three AA differences at positions 3, 4 and 6 (FV-R versus YI-L). Remarkably, some significant changes to the functionality of the enzyme were spotted due to the amino acid substitutions. Occurrence of T17G (Leu to Arg, position 6 of the enzyme) in four isolates, where the aliphatic side chain very hydrophobic leucine was substituted with the basic side chain hydrophilic arginine, indicates, higher potential in utility modification. Similarly, the substitutions of the hydroxyl and basic side chain in serine and lysine with aliphatic and an acidic amide side chain in glycine and glutamate in three isolates with different arrangement, suggest functional alteration at positions 234 and 235 on the enzyme, respectively. A fifth SHV variant (01–013906) possessed these three AA differences at positions 3, 4 and 6 and an additional two differences at positions 234 and 235 (GE versus SK).

### Community vs. nosocomial

Although the number of community isolates was quite small (7 versus 58) compared to the nosocomial isolates, a higher ratio of community isolates carried plasmid-mediated ESBL genes. Fifty seven percent of the community isolates carried CTX-m gene versus 5% nosocomial isolates which had the gene. Moreover, SHV has been detected in 43% of the community isolates, versus 13% of the nosocomial isolates. The significant presence of plasmid-mediated genes among the community isolates indicates higher potential of exchanging virulence factors in the community than the relative possibility in hospitals. In fact, nosocomial outbreaks occur due to dominating closely related strains that are being transmitted among hospitalized patients within same hospital wards or units. Accordingly, the probability for wide genetic variations is narrowed in hospital infections, due to the absence of selective environmental pressures by specific genetic elements, unlike the case in community infections where the unconstrained use of antibiotics is driving the continual transfer of antibiotic resistance genes. Thus, suggests applying restrictions on antibiotic usage, and the necessity for advances and finding alternatives for currently available treatment options.

### Macro-restriction pattern typing using Pulsed-Field Gel Electrophoresis (PFGE)

PFGE analysis did not demonstrate an association between genetic background and any given ESBL phenotype. Furthermore, as the macro-restriction pattern of SHV displays different patterns among the 11 *bla*shv positive isolates, it suggests possible carriage of the gene on different antibiotic resistance plasmids [24]. Such a variety of plasmids gives endless options to exchange virulence factors like antibiotic resistance, and hence a high potential of evolution.

In conclusion, this study demonstrates that CTX-m-15, and SHV-12 genes are associated with an ESBL resistance phenotype in *K. pneumoniae* in Egypt, without displaying a coherent related pattern of a DNA fingerprint profile. This suggests that the strains are heterogeneous, and their dissemination, whether in the community or at a hospital, was driven by strains of different genetic background that suggests great possibilities of variation and spread. Although one isolate (01–007967) conferred high ESBL resistance phenotype, it failed to identify any of the three genes reported in this study. Further investigation is required to determine the prevalence of such isolates in the community and hospital settings on a large scale and to identify other possible ESBL resistance mechanisms.

It is recommended that future work be done to determine the plasmid types that carried the plasmid-mediated genes mentioned, and apply the multilocus sequence typing (MLST) [13] to characterize the allelic profile of *bla*SHV *K. pneumoniae* isolates circulating in Egypt.

One limitation of the study is that we did not test for the horizontal movement of genes by performing a conjugation experiment to examine the self-transferable capability [25].

## Conclusions

This is the first study demonstrated the occurrence of SHV-12, and SHV-5 and SHV-2a variants in Egypt. SHV-12 is associated with high-level resistance to Ceftazidime, and one of the common class A ESBLs types spreading in K. pneumonia.

## Competing interests

The authors declared that they have no competing interest.

## Authors’ contributions

EAN carried out the molecular genetic studies, participated in the sequence alignment and drafted the manuscript. EAN, SFA participated in the sequence alignment. EAN, SFA, GP, BH, EV participated in the design of the study and performed the statistical analysis. EAN, SFA, GP, BH, EV conceived of the study, and participated in its design and coordination and helped to draft the manuscript. All authors read and approved the final manuscript.

## Supplementary Material

Additional file 1: Table S1 SHV amino acid sequence variations of ESBL Egyptian isolates. Click here for file
