# Supplementary material for: Detection of new SHV-12, SHV-5 and SHV-2a variants of extended spectrum Beta-lactamase in Klebsiella pneumoniae in Egypt
Source: Ann Clin Microbiol Antimicrob. 2013 Jul 18;12:16. doi: 10.1186/1476-0711-12-16 (PMC3723734; doi:10.1186/1476-0711-12-16)
Supplement: Additional file 1: Table S1 — SHV amino acid sequence variations of ESBL Egyptian isolates. [file 1476-0711-12-16-S1.pdf]

Additional File 1. SHV amino acid sequence variations of ESBL Egyptian isolates table.

```
               ....|....| ....|....| ....|....| ....|....| ....|....| ....|....|
                   5         15         25         35         45         55
02-000429|     MRYIRRCIIS LLATLPLAVH ASPQPLEQIK QSESQLSGRV GMIEMDLASG RTLTAWRADE
01-016401|     MRYIRRCIIS LLATLPLAVH ASPQPLEQIK LSESQLSGRV GMIEMDLASG RTLTAWRADE
03-002582|     MRYIRRCIIS LLATLPLAVH ASPQPLEQIK QSESQLSGRV GMIEMDLASG RTLTAWRADE
03-018785|     MRYIRRCIIS LLATLPLAVH ASPQPLEQIK LSESQLSGRV GMIEMDLASG RTLTAWRADE
01-007724|     MRYIRRCIIS LLATLPLAVH ASPQPLEQIK QSESQLSGRV GMIEMDLASG RTLTAWRADE
01-007743|     MRYIRLCIIS LLATLPLAVH ASPQPLEQIK QSESQLSGRV GMIEMDLASG RTLTAWRADE
01-007951|     MRFVRRCIIS LLATLPLAVH ASPQPLEQIK QSESQLSGRV GMIEMDLASG RTLTAWRADE
01-008666|     MRFVRRCIIS LLATLPLAVH ASPQPLEQIK QSESQLSGRV GMIEMDLASG RTLTAWRADE
01-013906|     MRFVRRCIIS LLATLPLAVH ASPQPLEQIK LSESQLSGRV GMIEMDLASG RTLTAWRADE
01-018867|     MRFVRRCIIS LLATLPLAVH ASPQPLEQIK QSESQLSGRV GMIEMDLASG RTLTAWRADE
03-021322|     MRFVRRCIIS LLATLPLAVH ASPQPLEQIK QSESQLSGRV GMIEMDLASG RTLTAWRADE
SHV-12 GB      MRYIRLCIIS LLATLPLAVH ASPQPLEQIK QSESQLSGRV GMIEMDLASG RTLTAWRADE
SHV-5 GB H     MRYIRLCIIS LLATLPLAVH ASPQPLEQIK LSESQLSGRV GMIEMDLASG RTLTAWRADE
SHV-2a GB      MRYIRLCIIS LLATLPLAVH ASPQPLEQIK LSESQLSGRV GMIEMDLASG RTLTAWRADE

              ....|....| ....|....| ....|....| ....|....| ....|....| ....|....|
                    65         75         85         95        105        115
02-000429|     RFPMMSTFKV VLCGAVLARV DAGDEQLERK IHYRQQDLVD YSPVSEKHLA DGMTVGELCA
01-016401|     RFPMMSTFKV VLCGAVLARV DAGDEQLERK IHYRQQDLVD YSPVSEKHLA DGMTVGELCA
03-002582|     RFPMMSTFKV VLCGAVLARV DAGDEQLERK IHYRQQDLVD YSPVSEKHLA DGMTVGELCA
03-018785|     RFPMMSTFKV VLCGAVLARV DAGDEQLERK IHYRQQDLVD YSPVSEKHLA DGMTVGELCA
01-007724|     RFPMMSTFKV VLCGAVLARV DAGDEQLERK IHYRQQDLVD YSPVSEKHLA DGMTVGELCA
01-007743|     RFPMMSTFKV VLCGAVLARV DAGDEQLERK IHYRQQDLVD YSPVSEKHLA DGMTVGELCA
01-007951|     RFPMMSTFKV VLCGAVLARV DAGDEQLERK IHYRQQDLVD YSPVSEKHLA DGMTVGELCA
01-008666|     RFPMMSTFKV VLCGAVLARV DAGDEQLERK IHYRQQDLVD YSPVSEKHLA DGMTVGELCA
01-013906|     RFPMMSTFKV VLCGAVLARV DAGDEQLERK IHYRQQDLVD YSPVSEKHLA DGMTVGELCA
01-018867|     RFPMMSTFKV VLCGAVLARV DAGDEQLERK IHYRQQDLVD YSPVSEKHLA DGMTVGELCA
03-021322|     RFPMMSTFKV VLCGAVLARV DAGDEQLERK IHYRQQDLVD YSPVSEKHLA DGMTVGELCA
SHV-12 GB      RFPMMSTFKV VLCGAVLARV DAGDEQLERK IHYRQQDLVD YSPVSEKHLA DGMTVGELCA
SHV-5 GB H     RFPMMSTFKV VLCGAVLARV DAGDEQLERK IHYRQQDLVD YSPVSEKHLA DGMTVGELCA
SHV-2a GB      RFPMMSTFKV VLCGAVLARV DAGDEQLERK IHYRQQDLVD YSPVSEKHLA DGMTVGELCA
```

```
               ....|....| ....|....| ....|....| ....|....| ....|....| ....|....|
                  125        135        145        155        165        175
02-000429|     AAITMSDNSA ANLLLATVGG PAGLTAFLRQ IGDNVTRLDR WETELNEALP GDARDTTTPA
01-016401|     AAITMSDNSA ANLLLATVGG PAGLTAFLRQ IGDNVTRLDR WETELNEALP GDARDTTTPA
03-002582|     AAITMSDNSA ANLLLATVGG PAGLTAFLRQ IGDNVTRLDR WETELNEALP GDARDTTTPA
03-018785|     AAITMSDNSA ANLLLATVGG PAGLTAFLRQ IGDNVTRLDR WETELNEALP GDARDTTTPA
01-007724|     AAITMSDNSA ANLLLATVGG PAGLTAFLRQ IGDNVTRLDR WETELNEALP GDARDTTTPA
01-007743|     AAITMSDNSA ANLLLATVGG PAGLTAFLRQ IGDNVTRLDR WETELNEALP GDARDTTTPA
01-007951|     AAITMSDNSA ANLLLATVGG PAGLTAFLRQ IGDNVTRLDR WETELNEALP GDARDTTTPA
01-008666|     AAITMSDNSA ANLLLATVGG PAGLTAFLRQ IGDNVTRLDR WETELNEALP GDARDTTTPA
01-013906|     AAITMSDNSA ANLLLATVGG PAGLTAFLRQ IGDNVTRLDR WETELNEALP GDARDTTTPA
01-018867|     AAITMSDNSA ANLLLATVGG PAGLTAFLRQ IGDNVTRLDR WETELNEALP GDARDTTTPA
03-021322|     AAITMSDNSA ANLLLATVGG PAGLTAFLRQ IGDNVTRLDR WETELNEALP GDARDTTTPA
SHV-12 GB      AAITMSDNSA ANLLLATVGG PAGLTAFLRQ IGDNVTRLDR WETELNEALP GDARDTTTPA
SHV-5 GB H     AAITMSDNSA ANLLLATVGG PAGLTAFLRQ IGDNVTRLDR WETELNEALP GDARDTTTPA
SHV-2a GB      AAITMSDNSA ANLLLATVGG PAGLTAFLRQ IGDNVTRLDR WETELNEALP GDARDTTTPA


               ....|....| ....|....| ....|....| ....|....| ....|....| ....|....|
                  185        195        205        215        225        235
02-000429|     SMAATLRKLL TSQRLSARSQ RQLLQWMVDD RVAGPLIRSV LPAGWFIADK TGASERGARG
01-016401|     SMAATLRKLL TSQRLSARSQ RQLLQWMVDD RVAGPLIRSV LPAGWFIADK TGASKRGARG
03-002582|     SMAATLRKLL TSQRLSARSQ RQLLQWMVDD RVAGPLIRSV LPAGWFIADK TGASKRGARG
03-018785|     SMAATLRKLL TSQRLSARSQ RQLLQWMVDD RVAGPLIRSV LPAGWFIADK TGAGERGARG
01-007724|     SMAATLRKLL TSQRLSARSQ RQLLQWMVDD RVAGPLIRSV LPAGWFIADK TGASKRGARG
01-007743|     SMAATLRKLL TSQRLSARSQ RQLLQWMVDD RVAGPLIRSV LPAGWFIADK TGASKRGARG
01-007951|     SMAATLRKLL TSQRLSARSQ RQLLQWMVDD RVAGPLIRSV LPAGWFIADK TGASKRGARG
01-008666|     SMAATLRKLL TSQRLSARSQ RQLLQWMVDD RVAGPLIRSV LPAGWFIADK TGASKRGARG
01-013906|     SMAATLRKLL TSQRLSARSQ RQLLQWMVDD RVAGPLIRSV LPAGWFIADK TGAGERGARG
01-018867|     SMAATLRKLL TSQRLSARSQ RQLLQWMVDD RVAGPLIRSV LPAGWFIADK TGASKRGARG
03-021322|     SMAATLRKLL TSQRLSARSQ RQLLQWMVDD RVAGPLIRSV LPAGWFIADK TGASKRGARG
SHV-12 GB      SMAATLRKLL TSQRLSARSQ RQLLQWMVDD RVAGPLIRSV LPAGWFIADK TGASKRGARG
SHV-5 GB H     SMAATLRKLL TSQRLSARSQ RQLLQWMVDD RVAGPLIRSV LPAGWFIADK TGASKRGARG
SHV-2a GB      SMAATLRKLL TSQRLSARSQ RQLLQWMVDD RVAGPLIRSV LPAGWFIADK TGASKRGARG
```

```
              ....|....| ....|....| ....|....| ....|....| ....|.
                   245        255        265        275        285
02-000429|    IVALLGPNNK AERIVVIYLR DTPASMAERN QQIAGIGAAL IEHWQR
01-016401|    IVALLGPNNK AERIVVIYLR DTPASMAERN QQIAGIGAAL IEHWQR
03-002582|    IVALLGPNNK AERIVVIYLR DTPASMAERN QQIAGIGAAL IEHWQR
03-018785|    IVALLGPNNK AERIVVIYLR DTPASMAERN QQIAGIGAAL IEHWQR
01-007724|    IVALLGPNNK AERIVVIYLR DTPASMAERN QQIAGIGAAL IEHWQR
01-007743|    IVALLGPNNK AERIVVIYLR DTPASMAERN QQIAGIGAAL IEHWQR
01-007951|    IVALLGPNNK AERIVVIYLR DTPASMAERN QQIAGIGAAL IEHWQR
01-008666|    IVALLGPNNK AERIVVIYLR DTPASMAERN QQIAGIGAAL IEHWQR
01-013906|    IVALLGPNNK AERIVVIYLR DTPASMAERN QQIAGIGAAL IEHWQR
01-018867|    IVALLGPNNK AERIVVIYLR DTPASMAERN QQIAGIGAAL IEHWQR
03-021322|    IVALLGPNNK AERIVVIYLR DTPASMAERN QQIAGIGAAL IEHWQR
SHV-12 GB     IVALLGPNNK AERIVVIYLR DTPASMAERN QQIAGIGAAL IEHWQR
SHV-5 GB H    IVALLGPNNK AERIVVIYLR DTPASMAERN QQIAGIGAAL IEHWQR
SHV-2a GB     IVALLGPNNK AERIVVIYLR DTPASMAERN QQIAGIGAAL IEHWQR
```
